# Author Correction: Sulcal organization in the medial frontal cortex provides insights into primate brain evolution

**DOI:** 10.1038/s41467-020-17973-0

**Published:** 2020-08-06

**Authors:** Céline Amiez, Jérôme Sallet, William D. Hopkins, Adrien Meguerditchian, Fadila Hadj-Bouziane, Suliann Ben Hamed, Charles R. E. Wilson, Emmanuel Procyk, Michael Petrides

**Affiliations:** 1 Univ Lyon, Université Lyon 1, Inserm, Stem Cell and Brain Research Institute U1208, 69500 Bron, France; 2 Wellcome Integrative Neuroimaging Centre, Department of Experimental Psychology, University of Oxford, Oxford, OX1 3SR UK; 3 Department of Comparative Medicine, University of Texas MD Anderson Cancer Center, Bastrop, TX 78602 USA; 4 Laboratoire de Psychologie Cognitive, UMR7290, Université Aix-Marseille, CNRS, 13331 Marseille, France; 5 Station de Primatologie CNRS, UPS846, 13790 Rousset, France; 6 Brain & Language Research Institute, Université Aix-Marseille, CNRS, 13604 Aixen-Provence, France; 7 Integrative Multisensory Perception Action & Cognition Team (ImpAct), INSERM U1028, CNRS UMR5292, Lyon Neuroscience Research Center (CRNL), University of Lyon 1, 69500 Lyon, France; 8 Institut des Sciences Cognitives Marc Jeannerod, UMR5229, CNRS-Université Claude Bernard Lyon I, 69675 Bron, France; 9 Montreal Neurological Institute, Department of Neurology and Neurosurgery and Department of Psychology, McGill University, Montreal, QC H3A 2B4 Canada

**Keywords:** Neuroscience, Cognitive neuroscience

Correction to: *Nature Communications*10.1038/s41467-019-11347-x, published online 31 July 2019.

The original version of this Article contained an error in the Acknowledgements section. The grant number provided in the text “C.A. received funding from the French National Research Agency (ANR-18-CE32-0012-01)”, was incorrect. This text has now been corrected to “C.A. received funding from the French National Research Agency (ANR-18-CE37-0012-01)”, in both the both the PDF and HTML versions of the Article.

