# Supplementary material for: Sulcal organization in the medial frontal cortex provides insights into primate brain evolution
Source: Nat Commun. 2019 Jul 31;10:3437. doi: 10.1038/s41467-019-11347-x (PMC6668397; doi:10.1038/s41467-019-11347-x)
Supplement: Supplementary file 1 — Supplementary Information [file 41467_2019_11347_MOESM1_ESM.pdf]

Supplementary information

Title: Sulcal organization in the medial frontal cortex provides insights into primate brain evolution

Amiez et al.

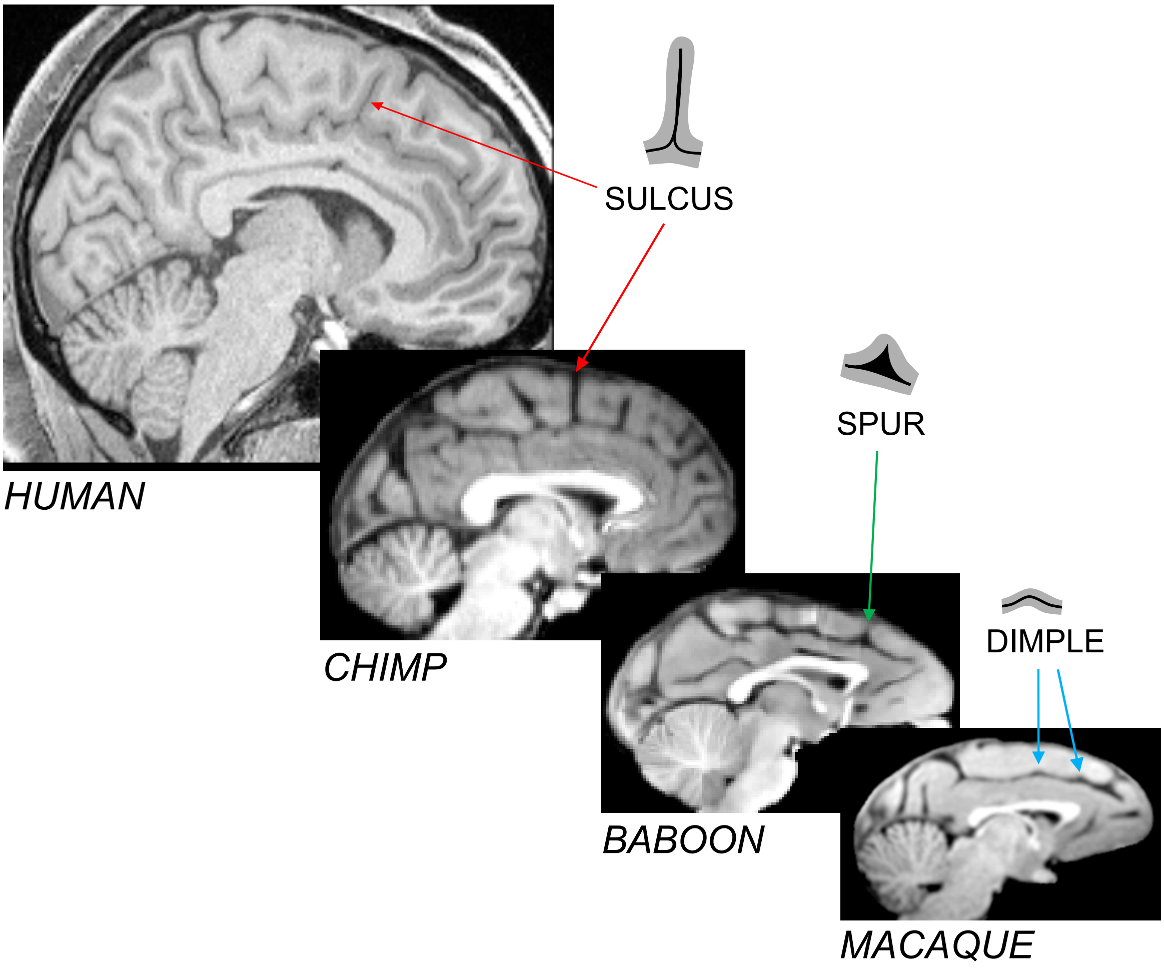

Supplemental Figure 1. Description of the vertical sulci in the MCC. Examples of the types of the vertical folds encountered in the mid-cingulate cortex (MCC). Vertical sulci are deep folds, i.e. fully formed sulci. Spurs and dimples are shallow folds encountered in this region mostly in baboon and macaque monkey brains.

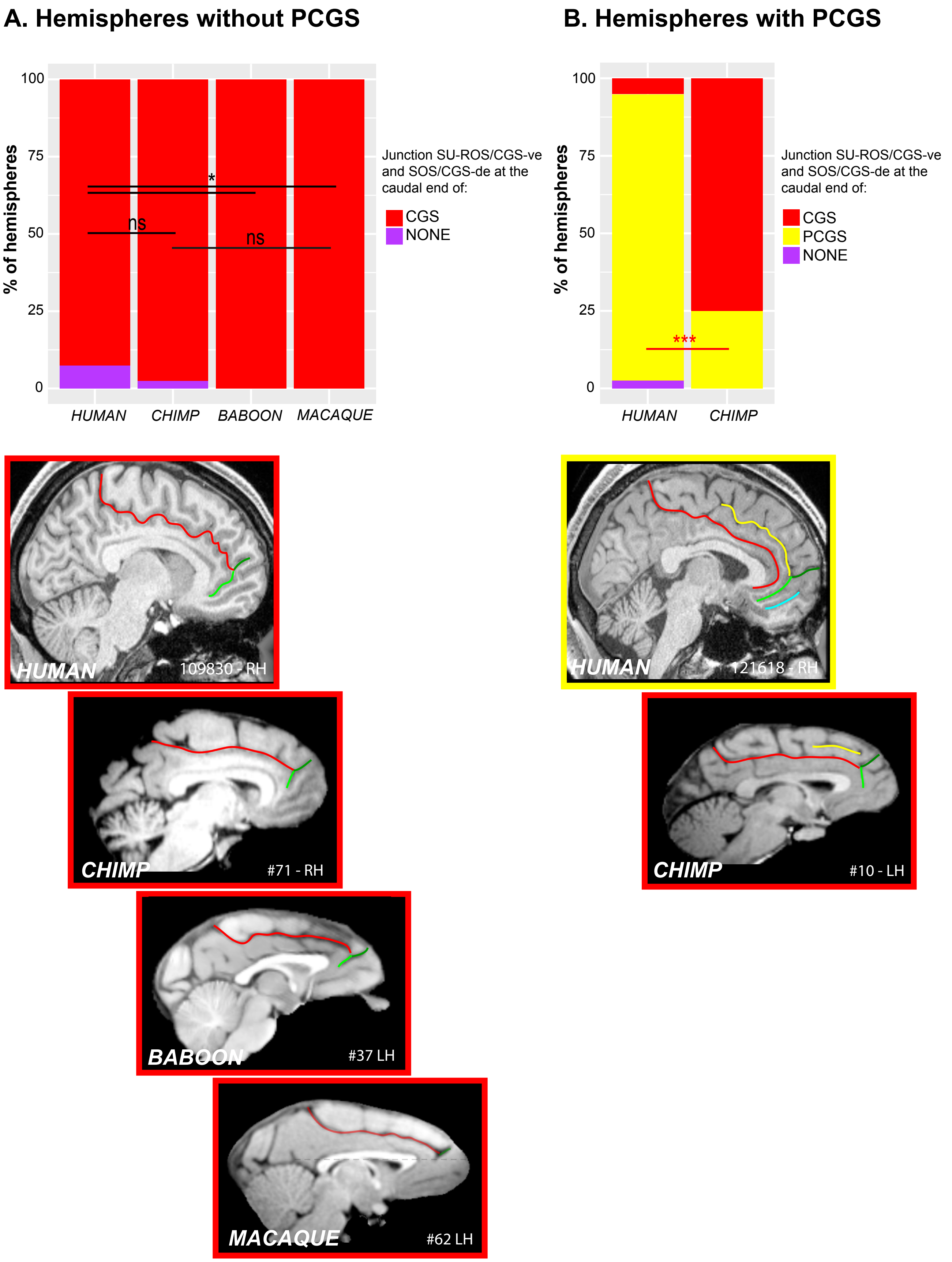

Supplemental Figure 2. Location of the SU-ROS/SOS and CGS-DE/CGS-VE intersection in hemispheres without (A) and with a PCGS (B) in primates. In human brains, when the PCGS is absent, the intersection is located at the rostral end of the CGS, but when the PCGS is present, it is located at the rostral end of the PCGS in the large majority of hemispheres. In chimpanzee, when the PCGS is absent, the intersection is located at the rostral end of the CGS. By contrast, when the PCGS is present, it is still located at the rostral end of the CGS in the large majority of hemispheres, and not of the PCGS as is the case in human brains.. In baboon and macaque, the intersection is located at the rostral end of the CGS. Source data are provided as a Source Data file.

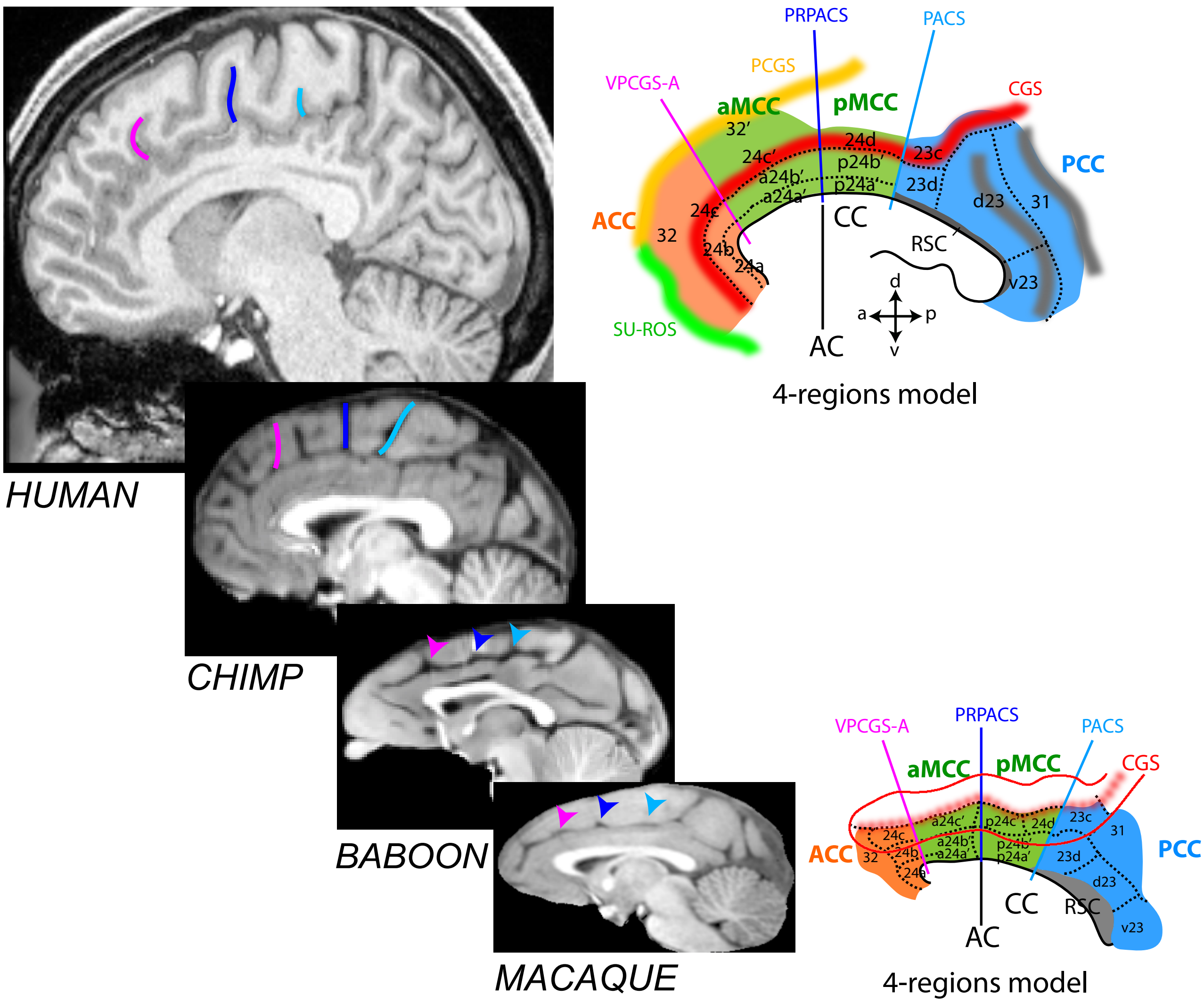

Supplemental Figure 3. Hypothetical relationships between the vertical folds in human and macaque medial frontal cortex (MFC) and cytoarchitectonic areas in the mid-cingulate cortex (MCC) from the 4-region model developed in both species. One hypothesis would be that the VPCGS-A is the limiting sulcus between the ACC and the aMCC in both human and macaque. Vogt's model identifies the limit between aMCC and pMCC as being the anterior commissure. Note that, as shown here, the PRPACS is systematically located at the level of the anterior commissure and is conserved across primates. Thus, this sulcus might be a limiting sulcus between aMCC and pMCC in both human and macaque. Finally, PACS that is located at the level of the rostral limit of the pons, might correspond to the limit between pMCC and PCC in both human and macaque. Since these three vertical sulci are conserved in baboon and in chimpanzee and display the same location as in the human and macaque brains, one can infer that they are limiting the same regions. Note that the 4-region model is represented on schematic views on the medial surfaces of the human and macaque brains. In the macaque brain, the cingulate sulcus (in red) is represented open with the dotted red line representing the fundus of the sulcus. Abbreviations: a, anterior; p, posterior; d, dorsal; v, ventral; AC, anterior commissure; cc, corpus callosum; MCC, mid-cingulate cortex; ACC, anterior cingulate cortex; PCC, posterior cingulate cortex; RSC, retrospenial cortex; CGS, cingulate sulcus; PCGS, paracingulate sulcus; PACS, paracentral sulcus; PRPACS, pre-paracentral sulcus; VPCGS-P and VPCGS-A, posterior and anterior vertical paracingulate sulcus, respectively; SU-ROS, supra-rostral sulcus.
