# Supplementary material for: Sulcal organization in the medial frontal cortex provides insights into primate brain evolution
Source: Nat Commun. 2019 Jul 31;10:3437. doi: 10.1038/s41467-019-11347-x (PMC6668397; doi:10.1038/s41467-019-11347-x)
Supplement: Supplementary file 3 — Reporting Summary [file 41467_2019_11347_MOESM3_ESM.pdf]

nature research

Corresponding author(s): AMIEZ CELINE

Last updated by author(s): 6/19/2019

Reporting Summary

Nature Research wishes to improve the reproducibility of the work that we publish. This form provides structure for consistency and transparency in reporting. For further information on Nature Research policies, see Authors & Referees and the Editorial Policy Checklist.

Statistics

For all statistical analyses, confirm that the following items are present in the figure legend, table legend, main text, or Methods section.

n/a	Confirmed	
☐	☒	The exact sample size (n) for each experimental group/condition, given as a discrete number and unit of measurement
☐	☒	A statement on whether measurements were taken from distinct samples or whether the same sample was measured repeatedly
☐	☒	The statistical test(s) used AND whether they are one- or two-sided<br>*Only common tests should be described solely by name; describe more complex techniques in the Methods section.*
☒	☐	A description of all covariates tested
☐	☒	A description of any assumptions or corrections, such as tests of normality and adjustment for multiple comparisons
☐	☒	A full description of the statistical parameters including central tendency (e.g. means) or other basic estimates (e.g. regression coefficient) AND variation (e.g. standard deviation) or associated estimates of uncertainty (e.g. confidence intervals)
☐	☒	For null hypothesis testing, the test statistic (e.g. F, t, r) with confidence intervals, effect sizes, degrees of freedom and P value noted<br>*Give P values as exact values whenever suitable.*
☒	☐	For Bayesian analysis, information on the choice of priors and Markov chain Monte Carlo settings
☒	☐	For hierarchical and complex designs, identification of the appropriate level for tests and full reporting of outcomes
☒	☐	Estimates of effect sizes (e.g. Cohen's d, Pearson's r), indicating how they were calculated

Our web collection on statistics for biologists contains articles on many of the points above.

Software and code

Policy information about availability of computer code

Data collection	NA
Data analysis	Normalization of primate brains was performed with SPM12 (https://www.fil.ion.ucl.ac.uk/spm/software/spm12/).<br>All statistics were performed with R software, R Development Core Team under R-Studio.

For manuscripts utilizing custom algorithms or software that are central to the research but not yet described in published literature, software must be made available to editors/reviewers. We strongly encourage code deposition in a community repository (e.g. GitHub). See the Nature Research guidelines for submitting code & software for further information.

Data

Policy information about availability of data

All manuscripts must include a data availability statement. This statement should provide the following information, where applicable:

- Accession codes, unique identifiers, or web links for publicly available datasets
- A list of figures that have associated raw data
- A description of any restrictions on data availability

Anatomical (T1) neuroimaging data of human and macaque brains are available from the Human Connectome Project (humanconnectome.org) and PRIME-DE (http://fcon_1000.projects.nitrc.org/indi/indiPRIME.html) databases, respectively. Chimpanzee anatomical data are available upon request to William Hopkins (http://www.chimpanzeebrain.org/). Baboon anatomical data are available upon request to Adrien Meguerditchian.

Field-specific reporting

Please select the one below that is the best fit for your research. If you are not sure, read the appropriate sections before making your selection.

☒ Life sciences ☐ Behavioural & social sciences ☐ Ecological, evolutionary & environmental sciences

For a reference copy of the document with all sections, see nature.com/documents/nr-reporting-summary-flat.pdf

Life sciences study design

All studies must disclose on these points even when the disclosure is negative.

Sample size	Qualitative analysis: Neuroimaging T1 structural data of 197 human brains (394 hemispheres), 225 chimpanzee brains (450 hemispheres), 88 baboon brains (176 hemispheres) and 80 macaque brains (160 hemispheres) were analyzed.<br>Quantitative analysis: Neuroimaging T1 structural data of 40 human brains (80 hemispheres), 40 chimpanzee brains (80 hemispheres), 40 baboon brains (80 hemispheres) and 40 macaque brains (80 hemispheres) were analyzed.
Data exclusions	No exclusion.
Replication	NA
Randomization	Randomization is not pertinent in our study. Human brains, chimpanzee brains, baboons brains and macaque brains are treated separately.
Blinding	Blinding across group is also not relevant, primate brains from different species being analysed separately.

Reporting for specific materials, systems and methods

We require information from authors about some types of materials, experimental systems and methods used in many studies. Here, indicate whether each material, system or method listed is relevant to your study. If you are not sure if a list item applies to your research, read the appropriate section before selecting a response.

Materials & experimental systems

n/a	Involved in the study	
☒	☐	Antibodies
☒	☐	Eukaryotic cell lines
☒	☐	Palaeontology
☐	☒	Animals and other organisms
☐	☒	Human research participants
☒	☐	Clinical data

Methods

n/a	Involved in the study	
☒	☐	ChIP-seq
☒	☐	Flow cytometry
☐	☒	MRI-based neuroimaging

Animals and other organisms

Policy information about studies involving animals; ARRIVE guidelines recommended for reporting animal research

Laboratory animals	Chimpanzee (pan troglodyte), baboon (papio papio), macaque (macacca mulatta)
Wild animals	None
Field-collected samples	NA
Ethics oversight	We did not aquire novel data in the present article. Rather, data correspond to existing databases in the laboratories of the authors involved. Note however that data collected initially for studies on macaque and baboons were conducted with local ethics agreements (licenses from the United Kingdom (UK) Home Office; Provence and Lyon ethic committees) and in accordance with The Animals (Scientific Procedures) Act 1986 and with the European Union guidelines (EU Directive 2010/63/EU). Chimpanzee data collection was approved by the Institutional Animal Care and Use Committees at YNPRC and UTMDACC and also followed the guidelines of the Institute of Medicine on the use of chimpanzees in research.

Note that full information on the approval of the study protocol must also be provided in the manuscript.

Human research participants

Policy information about studies involving human research participants

Population characteristics	Healthy human subjects from the Human Connectome Database.

Recruitment	The full set of inclusion and exclusion criteria is detailed in Van Essen et al. (2012). In short, the HCP subjects are healthy individuals who are free from major psychiatric or neurological illnesses. They are drawn from ongoing longitudinal studies, where they received extensive previous assessments including the history of drug use, emotional, and behavioral problems.
Ethics oversight	The experiments mentioned in the Human Connectome Database were performed in accordance with relevant guidelines and regulations and all experimental protocol was approved by the Institutional Review Board (IRB) (IRB # 201204036; Title: 'Mapping the Human Connectome: Structure, Function, and Heritability'). Furthermore, this project received approval (n° 15-213) from the ethic committee of Inserm (IORG0003254, FWA00005831) and from the Institutional Review Board (IRB00003888) of the French institute of medical research and health.

Note that full information on the approval of the study protocol must also be provided in the manuscript.

Magnetic resonance imaging

Experimental design

Design type	None
Design specifications	None
Behavioral performance measures	None

Acquisition

Imaging type(s)	Structural data came from existing databases in the various laboratories involved.
Field strength	1.5T and 3T
Sequence & imaging parameters	Structural sequences only were used.
Area of acquisition	Structural whole brain imaging

Diffusion MRI ☐ Used ☒ Not used

Preprocessing

Preprocessing software	spm12
Normalization	All primate structural data were normalized.
Normalization template	Human and macaque brains were normalized in the human (http://www.bic.mni.mcgill.ca/ServicesAtlases/HomePage) and macaque MNI steretotaxic coordinate system, respectively. Chimpanzee brains were normalized in the chimpanzee standard brain developed by Dr. W. Hopkins (Hopkins and Avants 2013, available at www.chimpanzeebrain.org). Baboon brains were normalized in the baboon standard brain developed by Dr. A. Meguerditchian (Love et al. 2016).
Noise and artifact removal	NA
Volume censoring	NA

Statistical modeling & inference

Model type and settings	NA
Effect(s) tested	NA

Specify type of analysis: ☒ Whole brain ☐ ROI-based ☐ Both

Statistic type for inference (See Eklund et al. 2016)	NA
Correction	NA

Models & analysis

n/a	Involved in the study
☒	☐ Functional and/or effective connectivity
☒	☐ Graph analysis
☒	☐ Multivariate modeling or predictive analysis
